# An Evaluation of the Tolerability and Feasibility of Combining 5-Amino-Levulinic Acid (5-ALA) with BCNU Wafers in the Surgical Management of Primary Glioblastoma

**DOI:** 10.3390/cancers13133241

**Published:** 2021-06-29

**Authors:** Colin Watts, Keyoumars Ashkan, Michael D. Jenkinson, Stephen J. Price, Thomas Santarius, Tomasz Matys, Ting Ting Zhang, Alina Finch, Peter Collins, Kieren Allinson, Sarah J. Jefferies, Daniel J. Scoffings, Athanasios Zisakis, Mark Phillips, Katharina Wanek, Paul Smith, Laura Clifton-Hadley, Nicholas Counsell

**Affiliations:** 1 Institute of Cancer and Genomic Sciences, University of Birmingham, Birmingham B15 2TT, UK; A.Finch.1@bham.ac.uk; 2 Department of Neurosurgery, Queen Elizabeth Hospital, Birmingham B15 2WB, UK; athaniasos.zisakis@uhb.nhs.uk; 3 Department of Neurosurgery, King’s College Hospital, London SE5 9RS, UK; k.ashkan@nhs.net; 4 Department of Neurosurgery, The Walton Centre, Liverpool L9 7LJ, UK; michael.jenkinson@liv.ac.uk; 5 Institute of Translational Medicine, University of Liverpool, Liverpool L69 3BX, UK; 6 Academic Neurosurgery Department, University of Cambridge, Cambridge CB2 0QQ, UK; sjp58@cam.ac.uk; 7 Department of Clinical Neurosciences, Cambridge University Hospitals Foundation Trust, Cambridge CB2 0QQ, UK; thomas.santarius@addenbrookes.nhs.uk (T.S.); vpc20@cam.ac.uk (P.C.); 8 Department of Radiology, Addenbrooke’s Hospital, Cambridge University Hospitals Foundation Trust, Cambridge CB2 0QQ, UK; tm418@cam.ac.uk (T.M.); tingtingzhang@doctors.org.uk (T.T.Z.); daniel.scoffings@addenbrookes.nhs.uk (D.J.S.); 9 Department of Histopathology, Cambridge University Hospitals Foundation Trust, Cambridge CB2 0QQ, UK; kieren.allinson@addenbrookes.nhs.uk; 10 Department of Oncology, Cambridge University Hospitals NHS Foundation Trust, Cambridge CB2 0QQ, UK; sarah.jefferies@addenbrookes.nhs.uk; 11 Cancer Institute, University College London, London WC1E 6DD, UK; mark.phillips12@nhs.net; 12 Cancer Research UK and University College London Cancer Trials Centre, London W1T 4TJ, UK; katharina_wanek@yahoo.co.uk (K.W.); p.smith56@btinternet.com (P.S.); l.clifton-hadley@ucl.ac.uk (L.C.-H.); nicholas.counsell@ucl.ac.uk (N.C.)

**Keywords:** glioblastoma, 5-aminolevulinic acid, BCNU wafers, chemoRT, feasibility trial

## Abstract

**Simple Summary:**

This reseach explored the safety and feasibility of combining local chemotherapy with fluorescence-guided resection in patients with a brain cancer, glioblastoma. The aim was to determine if the combination of fluorescence-guided surgery using 5-aminolevulinic acid and BCNU wafers left in the tumour cavity at the end of the operation was safe and did not prevent patients getting subsequent chemo-radiotherapy. The results showed that combining local chemotherapy with fluorescence-guided resection was tolerable in terms of surgical morbidity and overall toxicity. However, any potential therapeutic benefit requires further investigation, preferably with improved local delivery technologies.

**Abstract:**

*Background* Glioblastoma (GBM) is the commonest primary malignant brain tumour in adults and effective treatment options are limited. Combining local chemotherapy with enhanced surgical resection using 5-aminolevulinic acid (5-ALA) could improve outcomes. Here we assess the safety and feasibility of combining BCNU wafers with 5-ALA-guided surgery. *Methods* We conducted a multicentre feasibility study of 5-ALA with BCNU wafers followed by standard-of-care chemoradiotherapy (chemoRT) in patients with suspected GBM. Patients judged suitable for radical resection were administered 5-ALA pre-operatively and BCNU wafers at the end resection. Post-operative treatment continued as per routine clinical practice. The primary objective was to establish if combining 5-ALA and BCNU wafers is safe without compromising patients from receiving standard chemoRT. *Results* Seventy-two patients were recruited, sixty-four (88.9%) received BCNU wafer implants, and fifty-nine (81.9%) patients remained eligible following formal histological diagnosis. Seven (11.9%) eligible patients suffered surgical complications but only two (3.4%) were not able to begin chemoRT, four (6.8%) additional patients did not begin chemoRT within 6 weeks of surgery due to surgical complications. Eleven (18.6%) patients did not begin chemoRT for other reasons (other toxicity (*n* = 3), death (*n* = 3), lost to follow-up/withdrew (*n* = 3), clinical decision (*n* = 1), poor performance status (*n* = 1)). Median progression-free survival was 8.7 months (95% CI: 6.4–9.8) and median overall survival was 14.7 months (95% CI: 11.7–16.8). *Conclusions* Combining BCNU wafers with 5-ALA-guided surgery in newly diagnosed GBM patients is both feasible and tolerable in terms of surgical morbidity and overall toxicity. Any potential therapeutic benefit for the sequential use of 5-ALA and BCNU with chemoRT requires further investigation with improved local delivery technologies.

## 1. Introduction

Each year in the UK around 11,725 new cases of brain or central nervous system cancers are diagnosed, affecting around 7 per 100,000 of the population (source Cancer Research UK). The commonest of these is WHO Grade IV Astrocytoma, Glioblastoma (GBM), which accounts for over 80% of primary glial tumours. The median life expectancy in optimally managed patients is only 12–14 months with only 25% surviving 24 months [1]. The current clinical management of patients diagnosed with a GBM involves a combination of surgery, radiotherapy, and chemotherapy. However, survival trends for patients with GBM have remained largely static, reflecting the lack of therapeutic options for patients with these cancers [2]. Even in patients with a gross macroscopic resection, recurrence is the norm, with over 80% of relapsed disease occurring within 2 cm of the resection margin [3]. This observation has led to sustained interest in local therapies, particularly local chemotherapy [4].

Poly [carboxyphenoxy-propane/sebacic acid] anhydride wafers containing 3.85% carmustine-3-bis (2-choloroethyl 1)-1-Nitrosurea (BCNU) release carmustine over 2–3 weeks after being placed onto the surface of the tumour resection cavity [5]. BCNU wafers are the only local delivery technology approved for use in brain cancer patients. Initial clinical trial evaluation of local therapy with BCNU wafers in primary high-grade glioma showed promising improvement in survival [5,6]. However, these data were confounded by the inclusion of both WHO grade IV and grade III gliomas, which have very different survival profiles [7]. Subsequent long-term follow-up confirmed that grade III tumours dominated the long-term survivor cohort [8], and the use of BCNU wafers in the primary setting remains controversial [4,9].

Surgical advances have led to improved cytoreduction without compromising patient safety [10]. Improved cytoreduction in turn significantly improves clinical outcomes for GBM patients [11]. An important surgical adjunct that improves tumour resection is fluorescence-guided surgery using 5-aminolevulinic acid (5-ALA), which improves resection and progression-free survival [12]. A complete resection based on 5-ALA fluorescence can offer greater than 6-month OS in glioblastoma patients without an increase in post-operative neurological deficits [13,14].

The evaluation of local therapies in the context of current standard of care lacks prospective data [9]. The combination of fluorescence-guided resection using 5-ALA with local delivery of BCNU could enhance the benefit of subsequent chemoradiotherapy by optimizing disease control in the immediate post-operative period of 4–6 weeks until definitive treatment begins [7]. However, a prospective study to evaluate the safety, feasibility, and potential clinical benefit of combining 5-ALA and BCNU wafers in the surgical management of patients with newly diagnosed GBM has yet to be reported.

The development and evaluation of local delivery technologies has to take place in the context of the modern standard of clinical care. Therefore, it is necessary to understand the feasibility, tolerability, and safety of integrating BCNU wafers into the current pathway of care.

The aim of this clinical trial was to understand the impact of BCNU wafers on post-operative recovery and completion of chemoradiotherapy. We also sought to determine if the use of BCNU wafers was associated with increased surgical complications or new neurological deficits in patients undergoing 5-ALA resections. A secondary objective was to identify a signal of efficacy sufficient to support a randomized clinical trial.

## 2. Materials and Methods

### 2.1. Study Design and Treatment

This was a single arm study to evaluate the safety and tolerability of combining 5-ALA and BCNU wafers in the surgical management of patients with newly diagnosed GBM. The primary objective of the study was to identify any serious adverse impact of combining 5-ALA resection and BCNU-wafer insertion on post-operative recovery and completion of subsequent standard therapy. The secondary objective was to gather preliminary evidence of progression-free survival (PFS), overall survival (OS), and patient-reported QoL to inform the feasibility of an efficacy study.

Patients suspected of having a GBM based on magnetic resonance imaging (MRI) and judged suitable for radical resection at the neuro-oncology multi-disciplinary team (MDT) meetings (tumour board) were administered 5-ALA pre-operatively (up to 20 mg/kg, dissolved in tap water, 3–5 h prior to anaesthesia). Following intra-operative histological confirmation of “high-grade glioma likely GBM”, patients received BCNU-wafers after the tumour had been radically resected (up to eight discs, each containing 7.7 mg carmustine plus 192.3 mg polifeprosan 20). Subsequent radiotherapy and chemotherapy continued as per standard clinical practice [1].

Signed informed consent was obtained from all patients before any study-specific procedures were undertaken. Patient registration and trial management were performed by the Cancer Research United Kingdom and University College London Cancer Trials Centre (ClinicalTrials.gov identifier: NCT01310868, accessed on 21 June 2021).

### 2.2. Patients and Assessments

In addition to pre-operative MDT review, additional eligibility criteria were: age ≥18 years and WHO performance status of 0 or 1. Patients were excluded from trial participation if they had HIV, significant infection, or comorbidities that would preclude radical therapy; active liver disease; received concomitant anti-cancer therapy except steroids; a history of other malignancies within 5 years; undergone previous brain surgery or cranial radiotherapy; platelets < 100 × 10^9^/L; Mini-Mental State Examination score < 15; were pregnant or lactating or had evidence of contra-indication to 5-ALA or BCNU wafers. Eligibility assessments were completed a maximum of 2 weeks prior to registration.

Patients underwent a routine clinical MRI scanning protocol including contrast-enhanced MRI prior to the surgery, within 72 h of surgery, for radiotherapy planning, after three cycles of adjuvant temozolomide treatment, post-adjuvant temozolomide treatment (after six cycles), and then at 6-monthly intervals or upon symptomatic progression. MRI protocols varied between centres with regard to field strength (1.5 T or 3 T) and exact scanning parameters but included as a minimum T2, FLAIR, DWI, and pre- and post-contrast T1-weighted sequences in a matching plane. Images were not reviewed centrally prior to patient inclusion but were reviewed by two neuroradiologists upon study completion. Complete resection of enhancing tumour was defined as lack of any residual enhancement in the post-operative MRI (within 72 h). Radiological progression was defined as per RANO criteria [15].

Neurological examinations using NIH stroke score, surgical complications, adverse events, performance status, quality of life (QoL), and a record of the administration of steroids, other treatment, and concomitant medications were reported at baseline; within one month of surgery; pre-radiotherapy; pre-, mid-, and post-adjuvant chemotherapy; at 12-, 18-, and 24-months post-surgery; and then 6-monthly until death.

### 2.3. Genetic Analysis

Samples were tested for IDH1 and IDH2 mutations as follows: the IDH1 R132H mutation by immunostaining [16] followed, if negative, by pyrosequencing to identify if any of the less frequent mutations were present, i.e., IDH1 R132G, IDH1 R132C, IDH2 R172K, IDH2 R172M, and IDH2 R172W mutations [17]. The average MGMT methylation of CpGs 74–89 inclusive was determined by bisulphite modification of DNA followed by pyrosequencing. An average value over these 16 CpGs of greater than 10% was taken to indicate significant methylation [17].

### 2.4. Outcomes and Statistical Considerations

The primary objective was to establish that the combined use of 5-ALA and BCNU wafers did not compromise a patient from getting standard chemoRT. Assuming a target rate of ≥95% completing chemoRT, <85% of eligible patients completing chemoRT due to the combined use of 5-ALA and BCNU would be unacceptable. Using an exact sample size calculation, with one-sided 5% significance level and 80% power, 60 eligible patients receiving 5-ALA and BCNU wafers were required. The following endpoints were included to further assess safety and tolerability: procedure compliance; post-operative complication rate; failure to start, delays and interruptions to chemoRT due to surgical complications; WHO performance status before and after surgery. The secondary objective was to gather preliminary evidence of progression-free survival (PFS), overall survival (OS), and patient-reported QoL.

The outcome measures are presented using descriptive statistics. PFS and OS were analysed using the Kaplan–Meier method; patients were censored on the date they were last seen if no event had occurred. Analyses were carried out on all eligible patients, unless otherwise stated, and were generated using SAS software version 9.4 (SAS Institute Inc., Cary, NC, USA) and GraphPad Prism version 6 (GraphPad Software, La Jolla, CA, USA).

## 3. Results

### 3.1. Patients

Seventy-two patients were recruited from eight UK sites between July 2011 and May 2013. High suspicion of GBM was confirmed in sixty-two cases based on typical appearances of an intra-axial tumour with aggressive enhancement, surrounding T2-hyperintensity and no restricted diffusion within the enhancing component. Seven were classified as not typical for GBM, three of these were subsequently confirmed as GBM on histopathological analysis and four were other tumour types. Three patients did not have preoperative images available for central review.

Sixty-four (88.9%) of the seventy-two patients recruited received BCNU wafer implants; fifty-nine patients had an extent of resection ≥90%, one patient had 70%, and data were unavailable for four patients (neurosurgeon considered resection to be complete in all four cases). Eight patients (11.1%) did not receive BCNU wafer implants due to “high-grade glioma likely GBM” not being confirmed peri-operatively in four patients, ventricular breach in three patients, and peri-operative haemorrhage in one patient. A further four patients were ineligible due to GBM not being diagnosed post-operatively (anaplastic oligodendroglioma (*n* = 3, two grade 3 and one not specified); anaplastic astrocytoma (*n* = 1, grade 3)), and one patient had a simultaneous diagnosis of unrelated cutaneous sebaceous carcinoma.

**Figure 1:**
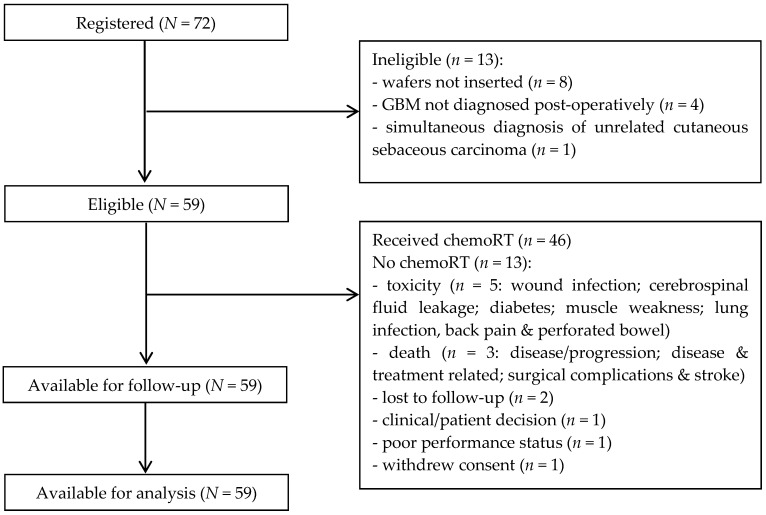
Consort diagram.

In total, 59 (81.9%) of the seventy-two patients recruited remained eligible to participate in the study after formal diagnosis (Figure 1). The characteristics of all eligible patients are shown in Table 1; median age was 59 years (range 37–71) and 37 (62.7%) were male.

### 3.2. Treatment Compliance and Safety

Seven (11.9%) of the fifty-nine eligible patients suffered surgical complications: wound infections were reported in five patients (8.5%) and cerebrospinal fluid (CSF) leakage in four patients (6.8%). Two patients (3.4%) were not able to begin chemoRT due to surgical complications: one wound infection and one CSF leakage. Other reasons patients were not able to start chemoRT were other toxicity (*n* = 3: diabetes; muscle weakness; lung infection, back pain, and perforated bowel); death (*n* = 3); lost to follow-up (*n* = 2); clinical decision (*n* = 1); consent withdrawn (*n* = 1); and poor performance status (*n* = 1).

In total, 46 (78.0%) of the fifty-nine eligible patients received chemoRT, including 4 (6.8%) patients whose further treatment was delayed beyond 6 weeks after surgery due to surgical complications: 3 wound infections and 1 CSF leakage. Radiotherapy was given over a median (range) of 30 (30–31) fractions, with a total dose of 60.0 (54.0–62.0) Gy. Temozolomide was given concomitantly with radiotherapy over 42 (1–48) days at 75.0 (75.0–75.0) mg/m^2^ per day. Concomitant chemoRT was interrupted in a total of 18 (39.1%) of the forty-six patients. Chemotherapy was interrupted in 13/46 (28.3%) patients due to toxicity (*n* = 11: bone marrow suppression (*n* = 4); thromboembolic event (*n* = 2); confusion; colon perforation; infection; dysphasia; oedema), logistical reasons (*n* = 1), and unknown (*n* = 1), and radiotherapy was interrupted in 12/46 (26.1%) patients due to toxicity (*n* = 4: bilateral retinal detachment; confusion; wound oedema; wound infection) and logistical reasons (*n* = 8).

Forty-three (93.5%) of the forty-six patients who received chemoRT continued to adjuvant chemotherapy, one patient progressed before starting, and two were lost to follow-up. Adjuvant chemotherapy was completed without interruption in 24/43 (55.8%) patients, with 19/43 (44.2%) patients unable to complete adjuvant chemotherapy without interruption due to: toxicity (*n* = 11), disease progression (*n* = 5), administrative failure (*n* = 2), and unknown (*n* = 1).

During the course of the study, 34 (57.6%) of the fifty-nine eligible patients reported 79 adverse events of maximum grade ≥ 3 (Table 2), the most common of which were muscle weakness and seizure, which were each reported in 5 patients (8.5%). None of these grade ≥3 adverse events were “likely” related to 5-ALA, whilst 7 events in 6 patients (10.2%) were at least “possibly” related to the BCNU wafers: wound infection (*n* = 2), sepsis (*n* = 2), cerebrospinal fluid leakage (*n* = 1), cerebral oedema (*n* = 1), and seizure (*n* = 1).

### 3.3. Efficacy

Long-term follow-up was collected for more than four years after the last patient was registered; 1 patient (1.7%) was alive without progression, 3 patients (5.1%) were alive having progressed, and 55 patients (93.2%) had died. Causes of death were disease/progression (*n* = 50), combination of disease and treatment related complications (*n* = 2), surgical complications and stroke (*n* = 1), septicaemia (*n* = 1), and one missing.

**Figure 2:**
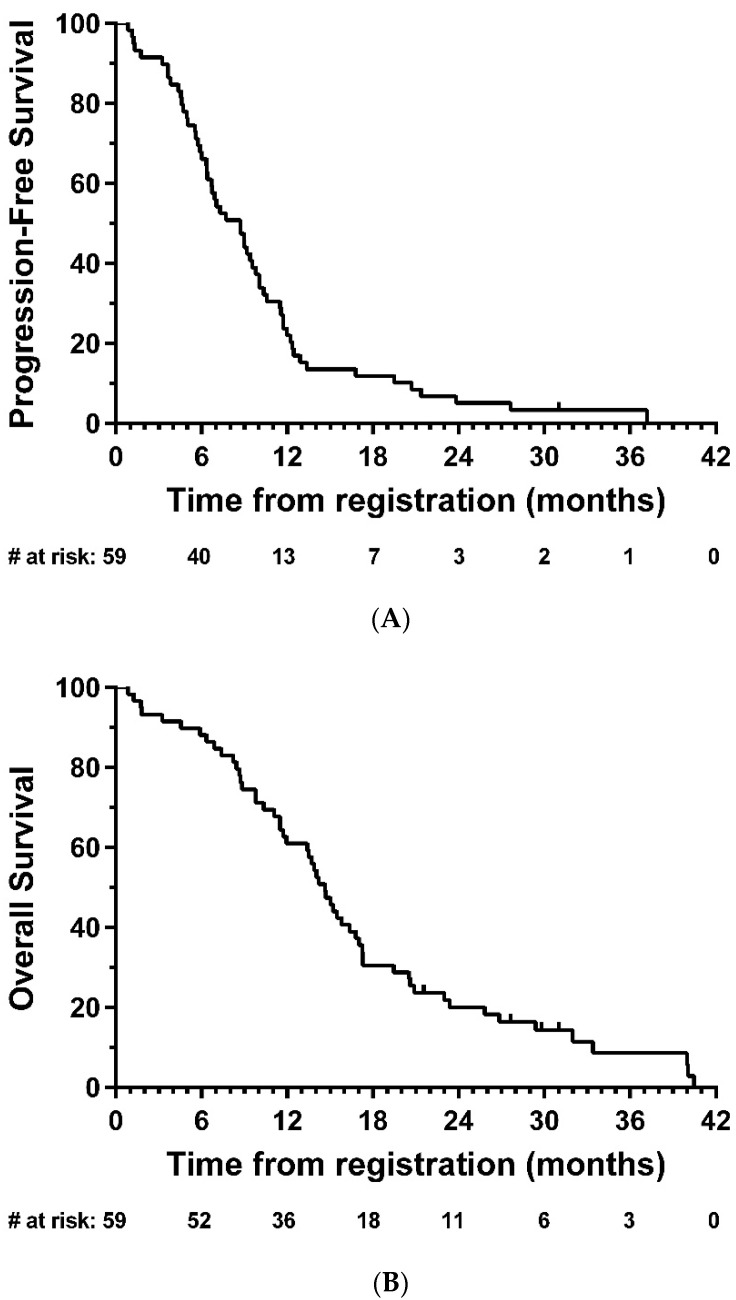
Kaplan–Meier plots of (**A**) progression-free and (**B**) overall survival—eligible patients. # = number.

**Figure 3:**
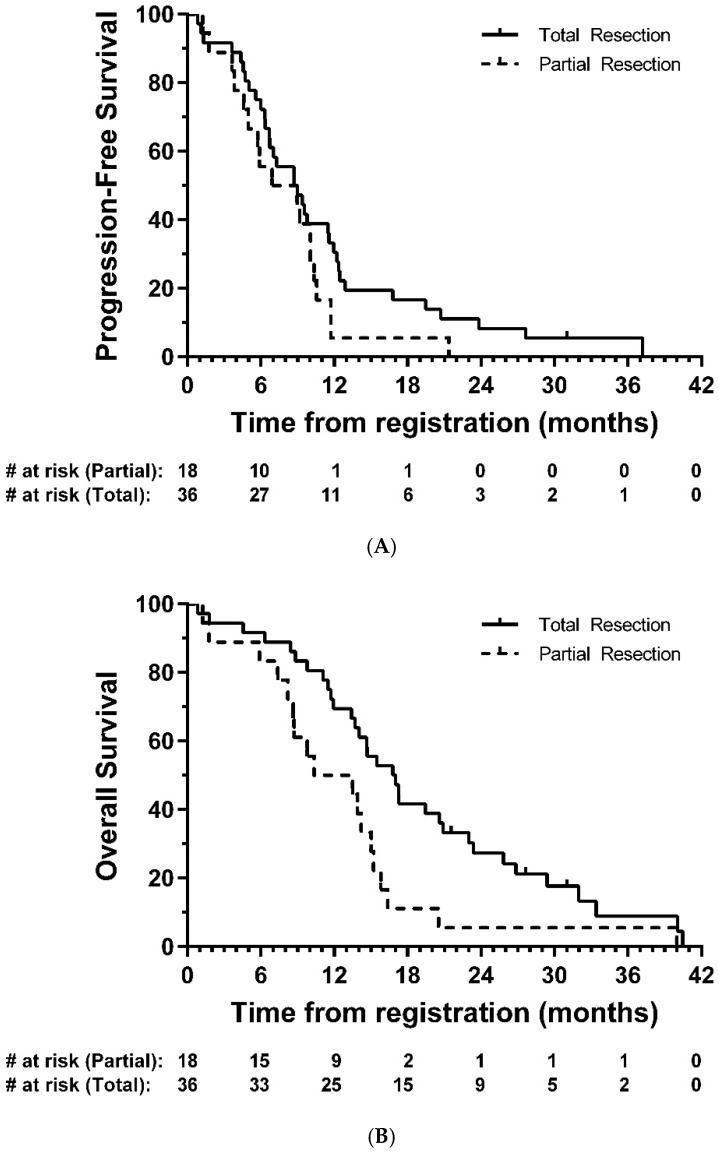
Kaplan–Meier plots of (**A**) progression-free and (**B**) overall survival—eligible patients, by resection group. # = number.

Median PFS was 8.7 months (95% CI: 6.4-9.8; Figure 2A), and median OS was 14.7 months (95% CI: 11.7-16.8, Figure 2B). In exploratory analyses, there was strong evidence of longer OS in the total resection group compared with partial resection (HR = 0.47, 95% CI: 0.26–0.85, *p* = 0.01); the same direction of effect was also observed for PFS, although this did not reach statistical significance (HR = 0.63, 95% CI: 0.35–1.14, *p* = 0.12) (Figure 3).

Compared with baseline WHO performance status, where available, 31 patients (31/57, 54.4%) were in the same category or improved post-surgery, and 26/57 (45.6%) had a lower WHO performance status post-surgery. In 6/57 (10.5%) post-surgery performance status was lower by more than one category.

There was a decrease in median Mini-Mental State Examination score between registration and post-adjuvant chemotherapy assessment (from a median of 28.0 to 26.0 points, *p* = 0.04; Table 3A) and an increase in median NIH Stroke Score (0.0 to 0.5 points, *p* = 0.001; Table 3B). There was no marked change in EORTC QoL functional domains over time, except for physical functioning (100.0 to 73.3 points, *p* < 0.001) and social functioning (83.3 to 66.7 points, *p* = 0.05), which decreased between registration and post-adjuvant chemotherapy assessment (Table 3C,D).

### 3.4. IDH1 and IDH2 Mutation and MGMT Methylation Analysis

**Figure 4:**
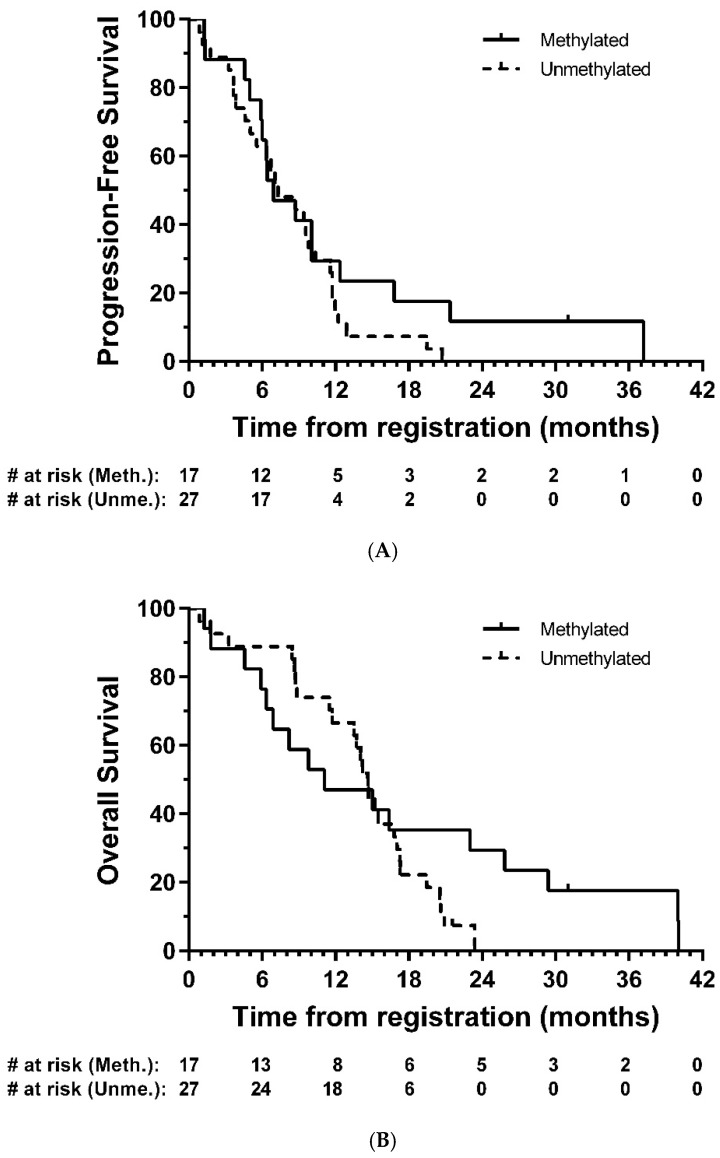
Kaplan–Meier plots of (**A**) progression-free and (**B**) overall survival—eligible patients, by MGMT methylation group. # = number.

Tumour tissue from forty-five patients were comprehensively analysed for IDH1 or IDH2 mutations and methylation of sixteen sites (CpG’s 74–89) in the CpG island of the MGMT gene. Only 1 (2.2%) of these was found to have an IDH1 mutation, the common R132H mutation; there were no other IDH1 or IDH2 mutations. Seventeen (37.8%) were considered to be methylated. In exploratory analyses, there was no strong evidence of a difference in PFS (HR = 0.67, 95% CI: 0.35–1.30, *p* = 0.24) or OS (HR = 0.65, 95% CI: 0.32–1.33, *p* = 0.24) between methylated and unmethylated groups, respectively (Figure 4).

## 4. Discussion

We report the results of a prospective single-arm feasibility study to evaluate the safety and tolerability of combining 5-ALA and BCNU wafers in the surgical management of patients with newly diagnosed GBM.

Of the seventy-two patients who underwent 5-ALA resection, 64 (88.9%) received BCNU wafers. Eight patients (11.1%) did not receive BCNU wafer implants due to “high-grade glioma likely GBM” not being confirmed peri-operatively in four patients, ventricular breach in three patients, and peri-operative haemorrhage in one patient. A further four patients were ineligible due to GBM not being diagnosed post-operatively (anaplastic oligodendroglioma (*n* = 3, two grade 3 and one not specified); anaplastic astrocytoma (*n* = 1, grade 3)). One patient had a simultaneous diagnosis of unrelated cutaneous sebaceous carcinoma. These exclusions meant that 59 (81.9%) of the seventy-two patients who underwent 5-ALA resection remained eligible.

Surgical complications of CSF leakage and wound infection were reported in 7 (11.9%) eligible patients. Grade 3 or higher adverse events possibly related to BCNU wafers were uncommon (2× wound infection, 2× sepsis, 1× CSF leakage, 1× cerebral oedema, 1× seizures), and there were no reports of intracranial hypertension.

In total, 46 (78.0%) of the eligible patients received chemoradiotherapy; 13 (22.0%) did not get further treatment, but only 2/59 (3.4%) were the result of using BCNU wafers. A further 4/59 (6.8%) were delayed starting further treatment due to surgical complications arising from BCNU wafers in 5-ALA resected GBM.

These data suggest that the use of 5-ALA combined with BCNU wafers had limited impact on patients receiving further treatment.

The morbidity associated with the use of BCNU wafers is variably reported in the literature. The available RCT data antedates the use of 5-ALA but reports no difference in cerebral oedema, wound healing, infections, seizures, and thromboembolic events between patients receiving BCNU wafers and controls [5,6,8,18]. Interestingly only CSF leaks (5% vs. 0.8%) and intracranial hypertension (9.1% vs. 1.7%) were significantly increased in patients receiving wafer implants in the study by Westphal et al. [5]. In a prospective observational surgical series of 113 GBM patients across 15 neurosurgical centres reflecting real-world practice in the UK, post-operative complications were reported in 23.6% (27/113), with surgical complications accounting for 48.1% (13/27) [19]. In this prospective study, the overall surgical complication rate was 11.9%, with CSF leakage in four patients and wound infection in five patients. These data are similar to several retrospective reports involving over 2300 patients [20,21,22,23]. In our study only 2 (3.4%) patients did not receive chemoRT due to surgical complications, which is again consistent with these retrospective studies.

However, treatment was interrupted in 18/46 (39.1%) patients who received concomitant chemoradiotherapy, due to toxicity or logistical reasons, and 19/43 (44.2%) of patients receiving adjuvant chemotherapy were unable to complete treatment without interruption, of which 11 (25.6%) were due to toxicity. These data are consistent with non-surgical trial data; in RTOG 0525 for example, 120/351 (34.2%) patients receiving standard adjuvant chemotherapy reported grade 3–5 toxicity [24]. Overall, our data suggests that the morbidity associated with intra-operative chemotherapy in the modern surgical era is acceptable and should not be used as a justification for withholding treatment or investing in research, including clinical trials.

The question of efficacy of BCNU wafers remains controversial. In this study we report a median PFS of 8.7 months and OS of 14.7 months, after the majority of patients (92.2%) who received BCNU wafers had >90% resection based on post-op MRI data. In exploratory analysis, a complete resection was associated with longer OS than that seen in patients with a partial resection (median 16.8 vs. 10.3 months, respectively). 

Several retrospective series reported improved survival from the use of BCNU wafers in the context of modern chemoradiotherapy and 5-ALA [21,22,23]. One study compared 5-ALA and BCNU versus control standard of care and concluded that there was synergy between enhanced resection using 5-ALA combined with the implantation of BCNU wafers [23]. Importantly, they also reported an increase in the number of patients surviving more than 3 years. Another reviewed 1659 high-grade glioma and reported a median survival of 18 months in newly diagnosed patients, with benefit from implanted wafers [22]. However, no distinction was made between anaplastic astrocytomas (WHO grade III) and GBM (WHO grade IV). Long-term follow-up of a cohort of GBM patients prospectively recruited into an RCT reported a trend for enhanced survival in BCNU-treated patients that did not reach significance [8]. In this RCT, of 11 patients alive after 56 months follow-up, 6 were Grade III astrocytomas, 2 were Grade IV astrocytomas, and 3 were “other” on histological diagnosis. In contrast, a retrospective evaluation of 5-ALA and BCNU in a UK cohort of 260 histologically confirmed GBM patients concluded that no residual disease, no residual fluorescence, and post-operative radio- and chemotherapy were all associated with improved outcome, but the use of BCNU wafers had no impact on survival [20].

The observed median PFS and OS of 8.7 months and 14.7 months here are similar to non-surgical studies in mixed MGMT methylated and unmethylated GBM populations using modern neuro-oncology protocols [24,25,26]. In an exploratory analysis, patients who were MGMT methylated generally displayed similar outcomes compared with those who were unmethylated despite a small number of patients in the methylated group having longer survival (>24 months in five patients). However, the sample size here is too small to draw definitive conclusions.

The use of BCNU wafers did not markedly impact performance status or quality of life (QoL) in this single-arm study. A decline in performance status in the early post-operative period was observed, but this was consistent with recovery from the surgical procedure itself [27]. There was no marked change in EORTC QoL functional domains over time, except for physical and social functioning, which decreased between registration and post-adjuvant chemotherapy assessment. These data are consistent with a retrospective analysis showing that BCNU wafers did not significantly reduce early post-operative KPS [21]. In the randomized-double blind phase III trial of Avastin in Glioblastoma (AVAglio) study (BO21990), the median time to deterioration in performance status was 6 months in the placebo arm and 9 months in the experimental arm [26]. The functional changes observed in our cohort of patients are consistent with those reported for high-grade glioma patients [28] and likely reflect a combination of treatment effect and disease progression rather than local delivery of chemotherapy [24].

An unexpected challenge in this study was rapid, accurate intra-operative diagnosis. The incidence of patients who could not be diagnosed as “high-grade glioma likely GBM” on frozen sections during surgery was 5.6% (4/72); these patients did not get wafers implanted. In patients who did receive BCNU wafers based on peri-operative tissue analysis, 6.3% (4/64) were re-diagnosed as anaplastic oligodendroglioma (*n* = 3) and anaplastic astrocytoma (*n* = 1). No patient had their wafers removed as a consequence of the change in diagnosis, but these examples highlight the importance of accurate diagnostic information in real-time during the operative procedure [29,30] in order to optimize surgical contribution to precision treatment in the context of local therapy [31].

The main limitation of this feasibility study was that the single-arm design does not allow for a direct comparison with a group of patients who did not receive the combination of 5-ALA and BCNU wafers. Additionally, the relatively moderate sample size means that evidence for therapeutic benefit is limited, and further investigation is required in a randomized setting. However, we provide prospective data with long-term follow-up in a multicentre trial setting which will help to inform the design of future studies. The nature of such research will need to recognize that perhaps the biggest issue with local delivery strategies for GBM is the characteristics of the BCNU wafers themselves. The biomaterial is stiff and releases the majority of its payload rapidly; this interferes with local wound healing, promoting the potential for CSF leakage and local infection.

**Figure 5:**
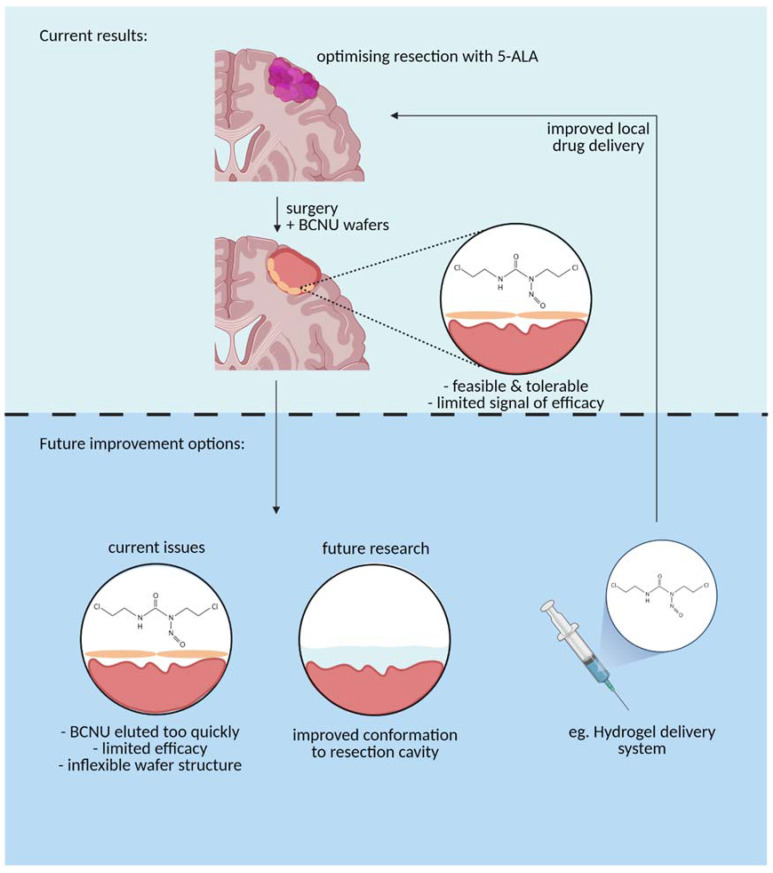
A graphical representation of the current BCNU drug delivery mechanism (**top panel**) vs. future improvements to the delivery mechanism to optimize use (**bottom panel**) (Created with BioRender.com, accessed on 13 April 2021).

The results of our study serve to emphasize that the clinical and biomaterials communities need to work together to deliver material that is more closely aligned with end-user requirements [32]. This has been discussed at length elsewhere [33], but a softer material that adheres to the irregular surface of the tumour cavity, elutes drug over a longer timescale, and is biodegradable would seem a reasonable starting point [34] (Figure 5). The engineering should be coupled with a clinical trial strategy for delivering results from the clinic to provide quantitative lessons and benchmarks that will guide the biomaterials community in developing novel local chemotherapy delivery systems.

## 5. Conclusions

- Use of 5-ALA and BCNU wafers in the surgical management of newly diagnosed GBM patients is both feasible and tolerable in terms of surgical morbidity and overall toxicity.
- Any potential therapeutic benefit of 5-ALA and BCNU wafers with chemoRT requires further investigation

## Figures and Tables

**Table 1:** Patient characteristics—eligible patients.

Patient Characteristic.	*N* = 59
	***n*** (%)
Gender	
Female	22 (37.3)
Male	37 (62.7)
Karnofsky Performance Status	
100	21 (35.6)
90	29 (49.2)
80	6 (10.2)
70	1 (1.7)
60	2 (3.4)
WHO Performance Status	
0	37 (62.7)
1 2–4	22 (37.3) 0 (0.0)
Tumour Location	
Frontal	20 (33.9)
Parietal	14 (23.7)
Temporal	14 (23.7)
Central	1 (1.7)
Occipital	1 (1.7)
Other: Frontal and Parietal	3 (5.1)
Other: Temporal and Parietal	3 (5.1)
Other: Parietal and Occipital	2 (3.4)
Other: Frontal and Temporal	1 (1.7)
Tumour Hemisphere	
Left	29 (49.2)
Right	29 (49.2)
Both	1 (1.7)
	**Median (Range)**
Age (years)	59.0 (37.0–71.0)
Haemoglobin (g/dL)	14.7 (11.7–17.7)
Platelets (×10^9^/L)	258.0 (125.0–475.0)
INR (*n* = 52)	0.9 (0.8–1.1)
Absolute Neutrophil Count (×10^9^/L) (*n* = 57)	11.1 (2.2–21.7)
White Blood Cell Count (×10^9^/L)	12.9 (4.9–25.3)

**Table 2:** Reported grade 3 or higher adverse events—eligible patients.

Adverse Events	*N* = 59	
	***n***	(%)
Any grade 3 or higher *	34	(57.6)
Any grade 3+ at least “possibly” related to 5-ALA	0	(0)
Any grade 3+ at least “possibly” related to BCNU wafers	6	(10.2)
Sepsis	2	(3.4)
Wound Infection	2	(3.4)
Cerebrospinal Fluid Leakage	1	(1.7)
Cerebral Oedema	1	(1.7)
Seizure	1	(1.7)
Any grade 3+ at least “possibly” related to surgery	15	(25.4)
Wound Infection	3	(5.1)
Muscle Weakness	2	(3.4)
Seizure	2	(3.4)
Thrombolytic Event	2	(3.4)
Blurred Vision	1	(1.7)
Cerebral Abscess	1	(1.7)
Cerebrospinal Fluid Leakage	1	(1.7)
Cerebral Oedema	1	(1.7)
Intra-operative Neurological Injury	1	(1.7)
Paraesthesia	1	(1.7)
Sepsis	1	(1.7)
Stroke	1	(1.7)
Visual Field Loss	1	(1.7)

* Grade 4 = 9 patients (15.3%); Grade 5 = 2 patients (3.4%).

**Table 3:** (**A**–**D**) Mini-Mental State Examination (**A**), NIH Stroke Scale (**B**), and Quality of Life (**C**,**D**)—eligible patients.

**(A) Mini-Mental State Examination (0–30, Severe to No Cognitive Impairment).**						
**Visit**					***N* = 59**	**Median (IQR)**
		
Registration					58	28.0 (25.0, 29.0)
Post-adjuvant chemotherapy					18	26.0 (22.0, 29.0)
		
Change from registration to post-adjuvant chemotherapy					18	−1.5 (−4.0, 2.0)
**(B) NIH Stroke Scale (0–42, No to Severe Symptoms).**						
**Visit**					***N* = 59**	**Median (IQR)**
Registration					58	0.0 (0.0, 1.0)
Post-surgery					54	0.0 (0.0, 1.0)
Pre-radiotherapy					39	0.0 (0.0, 2.0)
Post-radiotherapy					34	0.5 (0.0, 2.0)
Mid-adjuvant chemotherapy					26	1.0 (0.0, 2.0)
Post-adjuvant chemotherapy					18	0.5 (0.0, 2.0)
		
Change from registration to post-adjuvant chemotherapy					18	0.0 (0.0, 0.2)
**(C) Quality of Life: EORTC QLQ-C30 Functional Domains (0–100, No to High/Healthy Level of Functioning).**						
**Visit**	***N* = 59**	**Physical** **Functioning**	**Role** **Functioning**	**Emotional** **Functioning**	**Cognitive** **Functioning**	**Social** **Functioning**
Registration	58	100.0 (86.7, 100.0)	83.3 (50.0, 100.0)	79.2 (66.7, 91.7)	66.7 (66.7, 83.3)	83.3 (66.7, 100.0)
Pre-radiotherapy	36	80.0 (62.5, 93.3)	66.7 (33.3, 100.0)	83.3 (66.7, 95.8)	75.0 (66.7, 83.3)	66.7 (50.0, 83.3)
Post-radiotherapy	34	76.7 (53.3, 93.3)	66.7 (33.3, 100.0)	83.3 (66.7, 100.0)	66.7 (50.0, 83.3)	66.7 (33.3, 100.0)
Mid-adjuvant chemotherapy	29	73.3 (60.0, 93.3)	66.7 (33.3, 100.0)	83.3 (66.7, 100.0)	66.7 (50.0, 83.3)	66.7 (33.3, 100.0)
Post-adjuvant chemotherapy	20	73.3 (60.0, 86.7)	66.7 (33.3, 100.0)	75.0 (66.7, 100.0)	83.3 (66.7, 83.3)	66.7 (50.0, 100.0)
Change from registration to post-adjuvant chemotherapy	20	−20.0 (−40.0, 0.0)	0.0 (−33.3, 33.3)	0.0 (−20.8, 8.3)	0.0 (−16.7, 16.7)	−16.7 (−41.7, 0.0)
**(D) Quality of Life: EORTC QLQ-BN20 (0–100, No to Severe Symptoms).**						
**Visit**	***N* = 59**	**Future Uncertainty**	**Visual Disorder**	**Motor Dysfunction**		**Communication Deficit**
Registration	58	33.3 (16.7, 58.3)	0.0 (0.0, 22.2)	11.1 (0.0, 22.2)		16.7 (0.0, 33.3)
Pre-radiotherapy	36	25.0 (16.7, 50.0)	5.6 (0.0, 22.2)	22.2 (11.1, 33.3)		11.1 (0.0, 27.8)
Post-radiotherapy	34	29.2 (8.3, 50.0)	5.6 (0.0, 22.2)	16.7 (0.0, 33.3)		11.1 (0.0, 33.3)
Mid-adjuvant chemotherapy	29	16.7 (8.3, 41.7)	0.0 (0.0, 11.1)	11.1 (0.0, 22.2)		11.1 (0.0, 33.3)
Post-adjuvant chemotherapy	18	16.7 (8.3, 41.7)	0.0 (0.0, 11.1)	11.1 (0.0, 33.3)		11.1 (0.0, 22.2)
Change from registration to post-adjuvant chemotherapy	18	−8.3 (−25.0, 16.7)	0.0 (0.0, 0.0)	0.0 (0.0, 22.2)		0.0 (0.0, 11.1)

## Data Availability

The data are available on EudracT https://www.clinicaltrialsregister.eu/ctr-search/trial/2010-022496-66/results (accessed on 21 June 2021). Any access to data should be requested from the Cancer Research United Kingdom and University College London Cancer Trials Centre.

## References

[1] Stupp R., Mason W.P., van den Bent M.J., Weller M., Fisher B., Taphoorn M.J.B., Belanger K., Brandes A.A., Marosi C., Bogdahn U. (2005). Radiotherapy plus Concomitant and Adjuvant Temozolomide for Glioblastoma. N. Engl. J. Med..

[2] Brodbelt A., Greenberg D., Winters T., Williams M., Vernon S., Collins V.P. (2015). Glioblastoma in England: 2007–2011. Eur. J. Cancer.

[3] Hochberg F.H., Pruitt A. (1980). Assumptions in the radiotherapy of glioblastoma. Neurology.

[4] Wait S.D., Prabhu R.S., Burri S.H., Atkins T.G., Asher A.L. (2015). Polymeric drug delivery for the treatment of glioblastoma. Neuro. Oncol..

[5] Westphal M., Hilt D.C., Bortey E., Delavault P., Olivares R., Warnke P.C., Whittle I.R., Jääskeläinen J., Ram Z. (2003). A phase 3 trial of local chemotherapy with biodegradable carmustine (BCNU) wafers (Gliadel wafers) in patients with primary malignant glioma. Neuro. Oncol..

[6] Valtonen S., Timonen U., Toivanen P., Kalimo H., Kivipelto L., Heiskanen O., Unsgaard G., Kuurne T. (1997). Interstitial chemotherapy with carmustine-loaded polymers for high- grade gliomas: A randomized double-blind study. Neurosurgery.

[7] Weller M., van den Bent M., Tonn J.C., Stupp R., Preusser M., Cohen-Jonathan-Moyal E., Henriksson R., Rhun E.L., Balana C., Chinot O. (2017). European Association for Neuro-Oncology (EANO) guideline on the diagnosis and treatment of adult astrocytic and oligodendroglial gliomas. Lancet Oncol..

[8] Westphal M., Ram Z., Riddle V., Hilt D., Bortey E. (2006). Gliadel^®^ wafer in initial surgery for malignant glioma: Long-term follow-up of a multicenter controlled trial. Acta Neurochir..

[9] Ashby L.S., Smith K.A., Stea B. (2016). Gliadel wafer implantation combined with standard radiotherapy and concurrent followed by adjuvant temozolomide for treatment of newly diagnosed high-grade glioma: A systematic literature review. World J. Surg. Oncol..

[10] Sanai N., Berger M.S. (2018). Surgical oncology for gliomas: The state of the art. Nat. Rev. Clin. Oncol..

[11] Brown T.J., Brennan M.C., Li M., Church E.W., Brandmeir N.J., Rakszawski K.L., Patel A.S., Rizk E.B., Suki D., Sawaya R. (2016). Association of the extent of resection with survival in glioblastoma a systematic review and meta-Analysis. JAMA Oncol..

[12] Stummer W., Pichlmeier U., Meinel T., Wiestler O.D., Zanella F., Reulen H.J. (2006). Fluorescence-guided surgery with 5-aminolevulinic acid for resection of malignant glioma: A randomised controlled multicentre phase III trial. Lancet Oncol..

[13] Pichlmeier U., Bink A., Schackert G., Stummer W. (2008). Resection and survival in glioblastoma multiforme: An RTOG recursive partitioning analysis of ALA study patients. Neuro. Oncol..

[14] Hervey-Jumper S.L., Berger M.S. (2016). Maximizing safe resection of low- and high-grade glioma. J. Neurooncol..

[15] Wen P.Y., Macdonald D.R., Reardon D.A., Cloughesy T.F., Sorensen A.G., Galanis E., DeGroot J., Wick W., Gilbert M.R., Lassman A.B. (2010). Updated response assessment criteria for high-grade gliomas: Response assessment in neuro-oncology working group. J. Clin. Oncol..

[16] Capper D., Weißert S., Balss J., Habel A., Meyer J., Jäger D., Ackermann U., Tessmer C., Korshunov A., Zentgraf H. (2010). Characterization of r132h mutation-specific idh1 antibody binding in brain tumors. Brain Pathol..

[17] Collins V.P., Ichimura K., Di Y., Pearson D., Chan R., Thompson L.C., Gabe R., Brada M., Stenning S.P. (2014). Prognostic and predictive markers in recurrent high grade glioma; results from the BR12 randomised trial. Acta Neuropathol. Commun..

[18] Hart M.G., Garside R., Rogers G., Somerville M., Stein K., Grant R. (2011). Chemotherapy wafers for high grade glioma. Cochrane Database Syst. Rev..

[19] Ma R., Chari A., Brennan P.M., Alalade A., Anderson I., Solth A., Marcus H.J., Watts C., Kolias A., Sinha R. (2018). Residual enhancing disease after surgery for glioblastoma: Evaluation of practice in the United Kingdom. Neuro-Oncol. Pract..

[20] Sage W., Guilfoyle M., Luney C., Young A., Sinha R., Sgubin D., McAbee J.H., Ma R., Jefferies S., Jena R. (2018). Local alkylating chemotherapy applied immediately after 5-ALA guided resection of glioblastoma does not provide additional benefit. J. Neurooncol..

[21] Roux A., Peeters S., Zanello M., Bou Nassif R., Abi Lahoud G., Dezamis E., Parraga E., Lechapt-Zalcmann E., Dhermain F., Dumont S. (2017). Extent of resection and Carmustine wafer implantation safely improve survival in patients with a newly diagnosed glioblastoma: A single center experience of the current practice. J. Neurooncol..

[22] Champeaux C., Weller J. (2020). Implantation of carmustine wafers (Gliadel^®^) for high-grade glioma treatment. A 9-year nationwide retrospective study. J. Neurooncol..

[23] Della Puppa A., Lombardi G., Rossetto M., Rustemi O., Berti F., Cecchin D., Gardiman M.P., Rolma G., Persano L., Zagonel V. (2017). Outcome of patients affected by newly diagnosed glioblastoma undergoing surgery assisted by 5-aminolevulinic acid guided resection followed by BCNU wafers implantation: A 3-year follow-up. J. Neurooncol..

[24] Gilbert M.R., Wang M., Aldape K.D., Stupp R., Hegi M.E., Jaeckle K.A., Armstrong T.S., Wefel J.S., Won M., Blumenthal D.T. (2013). Dose-dense temozolomide for newly diagnosed glioblastoma: A randomized phase III clinical trial. J. Clin. Oncol..

[25] Gilbert M.R., Dignam J.J., Armstrong T.S., Wefel J.S., Blumenthal D.T., Vogelbaum M.A., Colman H., Chakravarti A., Pugh S., Won M. (2014). A Randomized Trial of Bevacizumab for Newly Diagnosed Glioblastoma. N. Engl. J. Med..

[26] Chinot O.L., Wick W., Mason W., Henriksson R., Saran F., Nishikawa R., Carpentier A.F., Hoang-Xuan K., Kavan P., Cernea D. (2014). Bevacizumab plus Radiotherapy–Temozolomide for Newly Diagnosed Glioblastoma. N. Engl. J. Med..

[27] Sacko A., Hou M.M., Temgoua M., Alkhafaji A., Marantidou A., Belin C., Mandonnet E., Ursu R., Doridam J., Coman I. (2015). Evolution of the Karnosky Performance Status throughout life in glioblastoma patients. J. Neurooncol..

[28] Armstrong T.S., Vera-Bolanos E., Acquaye A.A., Gilbert M.R., Ladha H., Mendoza T. (2016). The symptom burden of primary brain tumors: Evidence for a core set of tumor-and treatment-related symptoms. Neuro. Oncol..

[29] Shankar G.M., Francis J.M., Rinne M.L., Ramkissoon S.H., Huang F.W., Venteicher A.S., Akama-Garren E.H., Kang Y.J., Lelic N., Kim J.C. (2015). Rapid intraoperative molecular characterization of glioma. JAMA Oncol..

[30] Orringer D.A., Pandian B., Niknafs Y.S., Hollon T.C., Boyle J., Lewis S., Garrard M., Hervey-Jumper S.L., Garton H.J.L., Maher C.O. (2017). Rapid intraoperative histology of unprocessed surgical specimens via fibre-laser-based stimulated Raman scattering microscopy. Nat. Biomed. Eng..

[31] Shankar G.M., Kirtane A.R., Miller J.J., Mazdiyasni H., Rogner J., Tai T., Williams E.A., Higuchi F., Juratli T.A., Tateishi K. (2018). Genotype-targeted local therapy of glioma. Proc. Natl. Acad. Sci. USA.

[32] Gawley M., Almond L., Daniel S., Lastakchi S., Kaur S., Detta A., Cruickshank G., Miller R., Hingtgen S., Sheets K. (2020). Development and in vivo evaluation of Irinotecan-loaded Drug Eluting Seeds (iDES) for the localised treatment of recurrent glioblastoma multiforme. J. Control. Release.

[33] Tabet A., Jensen M.P., Parkins C.C., Patil P.G., Watts C., Scherman O.A. (2019). Designing Next-Generation Local Drug Delivery Vehicles for Glioblastoma Adjuvant Chemotherapy: Lessons from the Clinic. Adv. Healthc. Mater..

[34] Rowland M.J., Parkins C.C., McAbee J.H., Kolb A.K., Hein R., Loh X.J., Watts C., Scherman O.A. (2018). An adherent tissue-inspired hydrogel delivery vehicle utilised in primary human glioma models. Biomaterials.

